# ALBI Grade Is Associated with Clinical Outcomes of Critically Ill Patients with AKI: A Cohort Study with Cox Regression and Propensity Score Matching

**DOI:** 10.1155/2024/1412709

**Published:** 2024-07-18

**Authors:** Chao Yang, Zhikang Yu, Bo Peng, Changkun Mao, Junting Li, Yongsheng Cao

**Affiliations:** Department of Urology Anhui Provincal Children's Hospital, Hefei, China

## Abstract

**Background:**

The albumin–bilirubin (ALBI) grade has surfaced as a viable substitute for assessing liver functional reserve in individuals afflicted with hepatocellular carcinoma (HCC). ALBI grade also demonstrates the capacity to stratify distinct patient subcohorts bearing disparate prognostic implications in not only HCC but also other inflammatory diseases like acute pancreatitis. However, the association between ALBI grade and clinical outcomes of acute kidney injury (AKI) remains mysterious.

**Methods:**

The dataset was sourced from the Multiparameter Intelligent Monitoring in Intensive Care Database IV (MIMIC-IV) version 2.0. ALBI grade was calculated in a nomogram utilizing albumin and bilirubin. In order to ascertain the connection between ALBI grades and clinical outcomes of patients with AKI, Cox proportional hazards regression analysis was employed with in-hospital, 30- and 90-day mortality as end points, respectively. The Kaplan–Meier (K–M) curve was employed to gauge the cumulative incidence of mortality based on various ALBI grades. To explore potential nonlinear relationships, the Restricted Cubic Spline (RCS) approach was adopted. Furthermore, a subgroup analysis was conducted to validate the durability of the correlation between ALBI grade and in-hospital mortality. Furthermore, equilibrium of confounding variables was also achieved through the application of propensity score matching (PSM).

**Results:**

The study encompassed a total of 12,518 patients (ALBI grade 1 : 2878, grade 2 : 6708, and grade 3 : 2932). Patients with heightened ALBI grades displayed a significant correlation with increased mortality in both univariate and various multivariate Cox regression models. RCS depicted a predominantly linear relationship. The robustness of the correlation was also affirmed across multifarious subpopulations through subgroup analysis. The association still remains after PSM.

**Conclusion:**

Elevated ALBI grade was associated with worse clinical outcomes of critically ill patients with AKI.

## 1. Introduction

Acute kidney injury (AKI) is a severe clinical syndrome associated with significant morbidity and mortality [1, 2]. It is observed as a common comorbidity in critically ill patients [3], leading to prolonged hospital stays, increased costs, and a higher risk of in-hospital death as well as long-term mortality [4, 5]. Clinical and preclinical research has consistently demonstrated that the early detection and management of risk factors for AKI can effectively reduce in-hospital mortality [6, 7].

Albumin–Bilirubin (ALBI) grade was composed of two objective and readily available blood markers: serum albumin and total bilirubin. It was proposed in 2015 to evaluate liver function in patients with hepatocellular carcinoma [8]. It is reported that ALBI grade is capable of identifying different subgroups of patients with different prognoses and even predicting tumor relapse and postprocedural liver failure [9]. Furthermore, ALBI grade is also associated with inflammatory diseases, working as a prognostic biomarker for critically ill patients with acute pancreatitis [10] or HBV patients with cirrhosis [11].

The onset and development of AKI is closely associated with inflammatory response and its biomarkers [12, 13]. ALBI grade has been reported to be involved in AKI after platinum-based chemotherapy [14]. However, whether ALBI grade is associated with prognosis of critically ill patients with AKI remains unexplored.

Thus, we utilized large sample data from Multiparameter Intelligent Monitoring in Intensive Care Database IV (MIMIC-IV) to conduct this retrospective study, trying to reveal the association between ALBI grade and clinical outcome of critically ill patients with AKI.

## 2. Materials and Methods

### 2.1. Data Source

Information was gathered from version 2.0 of the MIMIC-IV, which encompassed over 60,000 ICU stays spanning from 2008 to 2019. The management of this database was conducted by the Beth Israel Deaconess Medical Center [15]. We successfully completed the protecting human research participants course, a web-based program offered by the National Institutes of Health. Our access to the database was granted by the Institutional Review Boards of both the Massachusetts Institute of Technology (Cambridge, MA, USA) and the Beth Israel Deaconess Medical Center.

### 2.2. Patient Selection

Patients received a diagnosis of AKI based on the Kidney Disease: Improving Global Outcomes (KDIGO) criteria. To be specific, this diagnosis was made when there was an elevation in serum creatinine by 0.3 mg/dL within a 48-hr period or an increase of 1.5 times from the baseline, as well as when urine output was less than 0.5 mL/kg/hr for a duration of 6 hr. The classification of AKI stages also considered serum creatinine (Scr) levels and urine volume in the initial 48 hr following admission to the ICU.

Patients were not considered for inclusion based on the subsequent criteria: (1) absence of albumin or bilirubin measurements before and after admission to the ICU, (2) age below 18 years, (3) expiration within 24 hr of ICU admission, (4) incomplete availability of clinical, or laboratory data records; and (5) instances of readmission.

### 2.3. Data Extraction

We employed Structured Query Language (SQL) to retrieve information recorded 24 hr before or after admission to the ICU. The gathered data encompassed details such as patients' gender and age, treatments administered such as ventilation and renal replacement therapy (RRT), scores such as the Simplified Acute Physiology Score (SAPS) II and the Sequential Organ Failure Assessment (SOFA) score, as well as comorbidities including malignant neoplasm of liver, other malignancy, cirrhosis of liver, sepsis, acute myocardial infarction (AMI), heart failure (HF), hypertension, chronic obstructive pulmonary disease (COPD), chronic kidney disease (CKD), atrial fibrillation (AF), cerebral infarction, cerebral hemorrhage, acute respiratory failure, rheumatic disease, and diabetes. Additionally, results from various laboratory tests including maximum of aspartate transaminase (AST), alkaline phosphatase (ASP), alanine transaminase (ALT), lactate, white blood cell (WBC) count, blood potassium, sodium, glucose, serum creatinine, blood urea nitrogen (BUN), international normalized ratio (INR), and minimum of blood calcium, platelet count, and hemoglobin were also enrolled. The survival time and status were also recorded. In-hospital mortality, 30-day mortality and 90-day mortality were also calculated.

### 2.4. Calculation of ALBI Grade

The initial values of albumin and bilirubin recorded 24 hr before or after admission to the ICU were utilized for calculation. The nomogram ((log10 bilirubin (in *μ*mol/L) × 0.66) + (albumin (in g/L) × −0.085)) was applied to calculate ALBI grade. There are three levels including grades 1, 2, and 3, respectively, corresponding to ≤−2.60, ≤−2.60 to ≤ −1.39, and >−1.39 [9]. Simultaneously, we also tried to divide patients into three groups based on quartile: 0%–25%, 25%–75%, 75%–100% and conducted further Cox regression.

### 2.5. Statistical Analysis

Continuous variables following a normal distribution were presented as mean ± standard deviation (SD), while categorical variables were reported as frequency (percent); one-way ANOVA or Student's *t*-test was employed to assess differences between groups for normally distributed continuous variables, whereas the Kruskal–Wallis test was used for non-normally distributed continuous variables, and the *χ*^2^ test was applied to examine categorical data.

In our study, Kaplan–Meier (K–M) curve, Cox proportional hazards regression model, and propensity score matching methodologies were utilized.

#### 2.5.1. Cox Proportional Hazards Regression

In Cox regression, patientswere categorized into three distinct groups as stated in calculation of ALBI grade. The quantitative values of laboratory tests were converted into categorical data by utilizing margins of the normal range or median. Specifically, AST, ALP, ALT, blood glucose, creatinine, BUN, INR, calcium, platelet, and hemoglobin were transformed into binary variables, while WBC, potassium, and sodium were classified into three levels. Among the former, glucose and creatinine were divided based on median considering the distribution of values.

We estimated the hazard ratio (HR) along with 95% confidence intervals (CIs) to examine the association between ALBI grade and in-hospital mortality, 30-day mortality and 90-day mortality, respectively. Patients with ALBI grade 1 were considered as the reference group in the Cox regression analysis. Three multivariate Cox models were constructed in this study. Model 1 accounted for age and gender as covariates. Model 2 further adjusted for comorbidities as stated in Section 2.3. Model 3 included additional adjustments for results from laboratory tests, SOFA and SPASII score, use of ventilation and RRT.

#### 2.5.2. Propensity Score Matching (PSM)

To ascertain the robustness of the correlation, we employed nearest PSM with a 1 : 1 ratio and a caliper of 0.005. Specifically, patients with ALBI grade 3 and patients with ALBI grade 2 were matched with patients of ALBI grade 1, respectively. Covariates which are likely to influence/reflect disease severity or clinical outcomes are matched in PSM. Subsequently, the mortality difference was evaluated using both the *χ*^2^ test and Cox regression after matching. Additionally, we also examined the potential nonlinear relationship between ALBI value and mortality by utilizing RCS curves.

We conducted a subgroup analysis to examine the association between ALBI grade and in-hospital mortality. We also evaluated the interactions of ALBI with factors included in Model 3 for adjustment.

The statistical analyses were carried out using R version 4.3.1 (R Foundation for Statistical Computing, Vienna, Austria) and Stata version 17.0 (StataCorp LLC, Texas, USA).

## 3. Results

### 3.1. Baseline of Population

A total of 12,518 eligible patients diagnosed with AKI were identified. Among our cohorts, 2,387 patients experienced mortality during the stay in hospital. The complete set of data mentioned in Materials and Methods has been extracted and is presented in Table 1.

**Figure 1:**
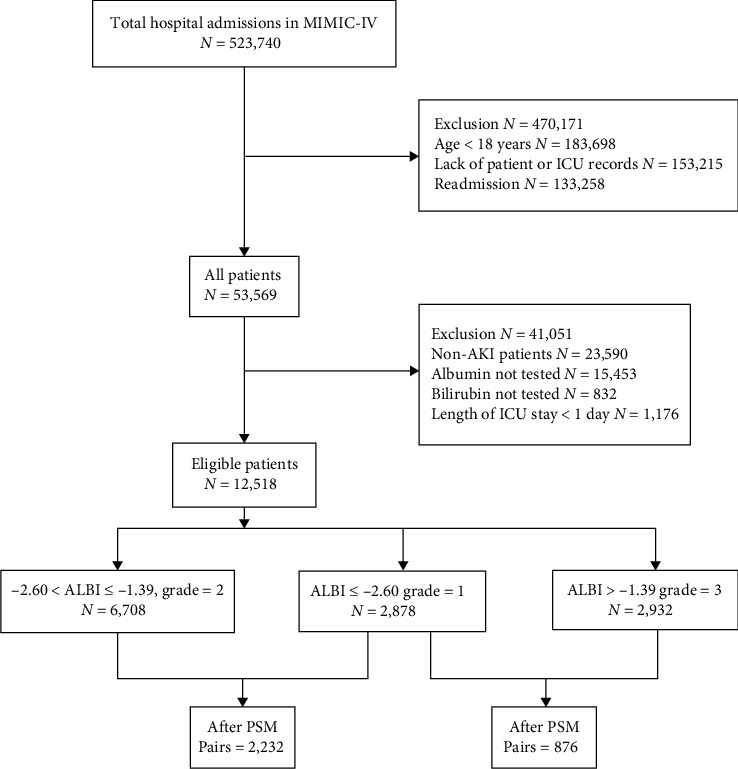
The flowchart of patient selection.

After PSM of ALBI grade 1 and grade 2 cohorts, 2,232 pairs of patients were left. As to ALBI grade 1 and grade 3 cohorts, 876 pairs of patients matched.  Table 2 demonstrates the balanced characteristics of the matched patients. The flowchart illustrating the process of patient selection is depicted in Figure 1.

### 3.2. ALBI and In-Hospital Mortality, 30-Day Mortality, and 90-Day Mortality

#### 3.2.1. K–M Survival Curve

**Figure 2:**
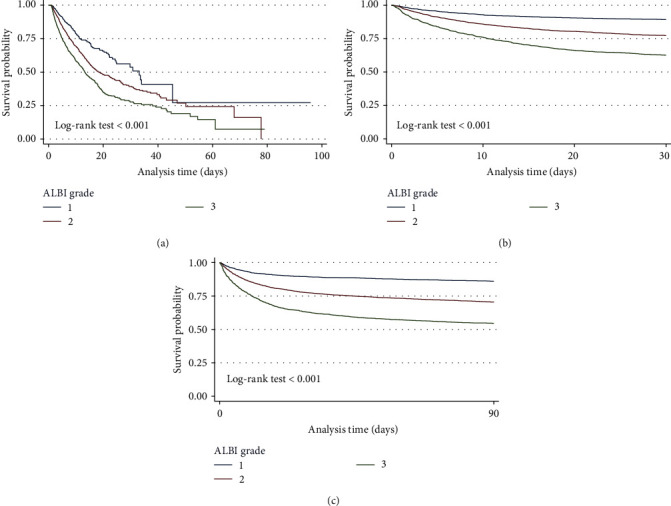
Kaplan–Meier survival curve for (a) in-hospital mortality, (b) 30-day mortality, and (c) 90-day mortality.

The K–M survival curve demonstrated noteworthy disparities in survival rates among three ALBI grades in in-hospital, 30- and 90-day mortality (log-rank test *P* < 0.001; Figure 2). The survival probability significantly declined with the elevation of ALBI grade.

#### 3.2.2. Cox Proportional Hazards Regression

The combined results of univariate and multivariate Cox regression are presented in Table 3, showcasing their aggregation. *Supplementary table 1* displays the outcomes of the variables adjusted for multivariate regression in Model 3.

In univariate Cox regression model, the HR and 95% CIs of ALBI grade 2 and grade 3 were, respectively, 2.34 (2.02, 2.70) and 5.17 (4.44, 6.02) for in-hospital mortality, then changed to 1.31 (1.11, 1.55) and 1.52 (1.24, 1.85) in Model 3. A similar trend can also be observed in the 30- and 90-day mortality. Elevated ALBI grade is always a risk factor for mortality in different models.

We also summarized the results of Cox regression if patients were divided by quartile of ALBI value. Similar trend can be seen in *Supplementary table 2*.

#### 3.2.3. Restricted Cubic Spline

**Figure 3:**
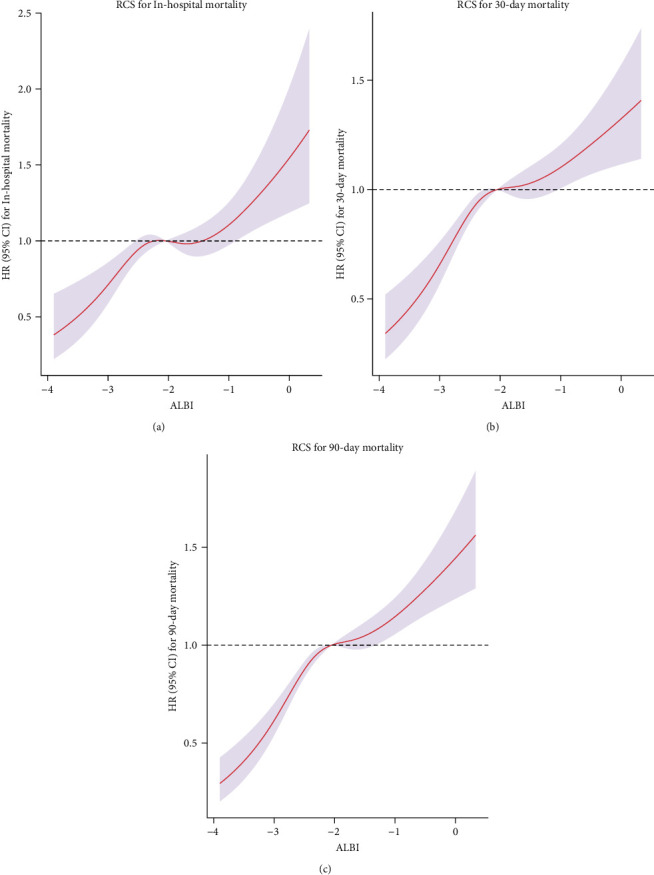
Restricted cubic spline curves for (a) in-hospital mortality, (b) 30-day mortality, and (c) 90-day mortality. The median (−2.05) of ALBI is used as cutoff line for HR = 1.

The Model 3-based RCS curve revealed a nearly linear relationship between ALBI value and in-hospital mortality, 30- and 90-day mortality (Figure 3).

### 3.3. Subgroup Analysis and *P* for Interaction

**Figure 4:**
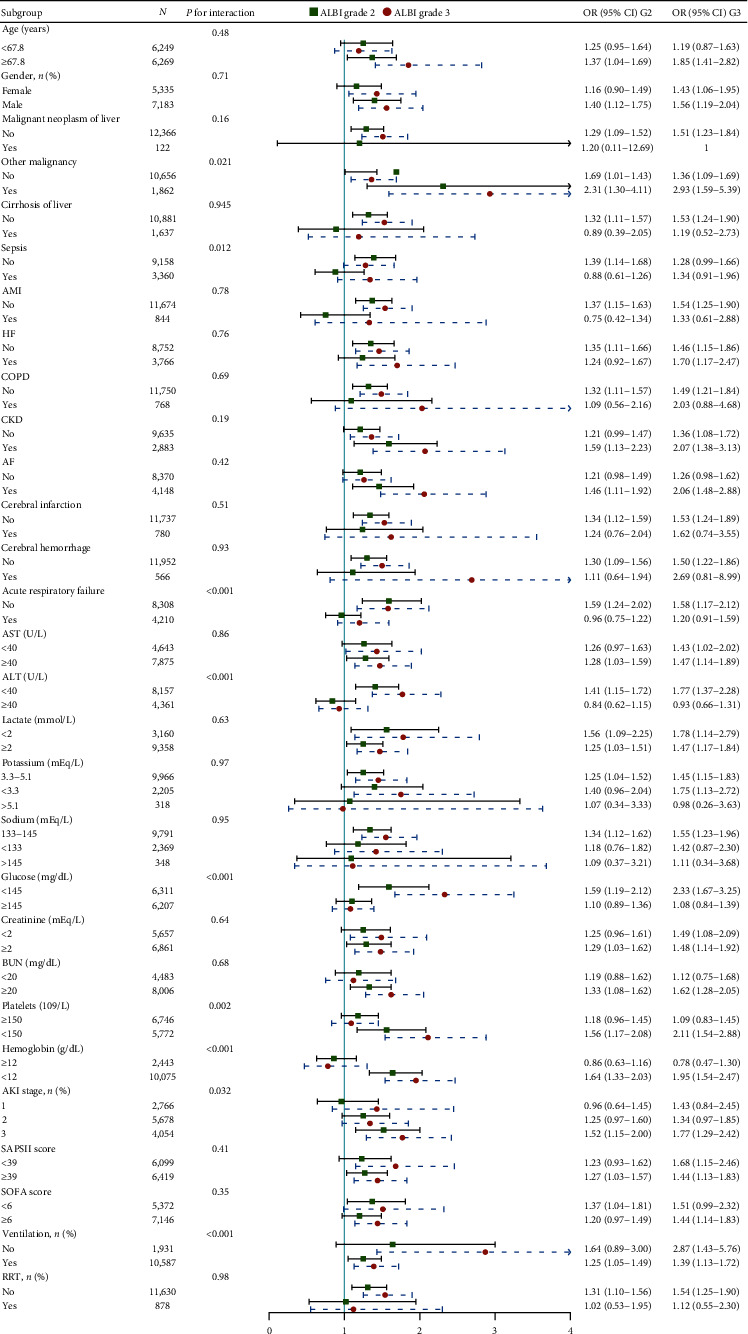
Forest plot showing subgroup analysis of Cox regression Model 3 for in-hospital mortality.

The majority of subpopulations exhibited comparable HR in relation to the correlation between fibrinogen and in-hospital mortality in Model 3. Sepsis, acute respiratory failure, ALT, blood glucose, platelets, hemoglobin, AKI stage, and utilization of ventilation significantly interacted with ALBI grade (Figure 4).

### 3.4. PSM

**Figure 5:**
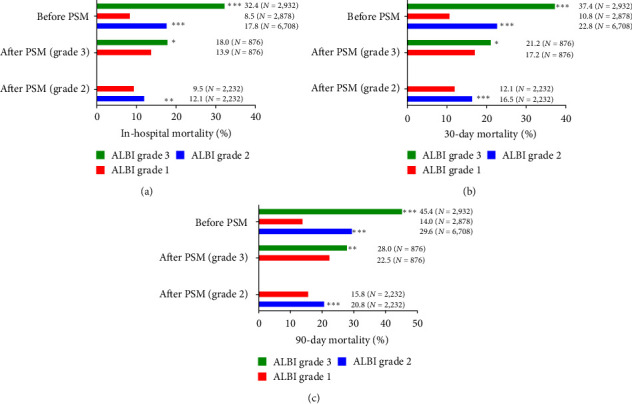
Summary of mortality and *χ*^2^ test before and after PSM: (a) In-hospital mortality, (b) 30-day mortality, and (c) 90-day mortality.  ^*∗*^*P* < 0.05,  ^*∗∗*^*P* < 0.01, and  ^*∗∗∗*^*P* < 0.001.

Statistical difference of mortality between ALBI grade 2/3 and grade 1 still exists in *χ*^2^ test after matching (Figure 5). We also conducted univariate Cox regression again, which still revealed that elevated ALBI grade is a risk factor for in-hospital, 30- and 90-day mortality of patients with AKI (Table 4).

## 4. Discussion

This large sample study suggests that elevated ALBI grade is associated with clinical outcomes (in-hospital mortality, 30-day mortality, and 90-day mortality) of critically ill patients with AKI. The K–M survival analysis and Cox regression both reveal significant difference in survival among patients with diverse ALBI grade. To be specific, patients with higher ALBI grade tend to have a higher risk of being deceased in hospital or 30- and 90 days after admission.

ALBI grade was initially proposed for assessment of hepatocellular carcinoma and related comorbidities or diseases [8]. Gradually, the application of ALBI was extended to acute upper gastrointestinal bleeding in liver cirrhosis [16, 17], acute pancreatitis [10], and even platinum-induced AKI [14]. Preoperative liver function was also associated with prognosis of cardiac surgery patients without liver disease [18]. Considering ALBI's close correlation with liver, we have to note the difference among diverse ALBI groups in baseline, especially ratio of patients with hepatic cirrhosis or carcinoma and liver enzymes. To be specific, patients with higher ALBI grade are accompanied by higher ratio of malignant neoplasm in liver, cirrhosis and elevated ALT and AST. Additionally, hepatorenal syndrome (HRS) also cause AKI in some critically ill patients [19]. To alleviate or eliminate potential bias induced by these situations, we include such covariates in multivariate Cox regression to adjust and adopt PSM to acquire pairs of patients with similar baseline. The results always support that elevated ALBI grade is an independent risk factor in AKI patients. Furthermore, we excluded patients with cirrhosis, elevated ALT or AST separately in subgroup analysis, HRs with 95% CIs are still significant for, as you can see in Figure 4.

Sepsis interacts significantly with ALBI grade in subgroup analysis (*P* for interaction <0.01). It was reported that sepsis was both inducing factor and result of AKI [20], which may be responsible for the significant interaction. Sepsis-associated acute kidney injury (S-AKI) may be an example of such an interrelation, which will lead to increased mortality and mutilation rate [21]. In our study, we also found that sepsis is a risk factor for clinical outcomes of AKI patients in multivariate Cox regression (in-hospital mortality, OR 1.50, 95% CI (1.33–1.68), *P* < 0.001; 30-day mortality, HR 1.17 (1.08–1.27), *P* < 0.001; and 90-day mortality 1.24 (1.15–1.34), *P* < 0.001). Moreover, according to subgroup analysis (Figure 4), in subpopulation without sepsis, elevation of ALBI grade is still a risk factor for in-hospital mortality.

Hemoglobin was also found to interact with ALBI grade (*P* for interaction <0.001) in multivariate Cox regression in Model 3. The accumulation of hemoglobin in proximal tubules was reported to deteriorate AKI induced by cisplatin or ischemic-reperfusion [22]. Patients with postoperative AKI also tend to be detected with higher cell-free hemoglobin than those without AKI [23]. Our results in subgroup analysis indicate that the association between ALBI grade and in-hospital mortality was more significant in patients with depressed hemoglobin (<12 g/dL). Although the level of blood hemoglobin cannot exactly reflect accumulated hemoglobin in kidney, the interaction between ALBI grade and hemoglobin may be partly attributed to the effect of hemoglobin on kidney injury.

Diabetes mellitus was reported to increase kidney susceptibility to AKI [24]. However, in our cohort, diabetes seems to be a protective factor for mortality, as shown in *Supplementary table 1*. Actually, in univariate regression for diabetes, it is not a significant factor (in-hospital mortality, OR 0.92, 95% CI (0.83–1.01), *P*=0.095; 30-day mortality, HR 0.96 (0.89–1.04), *P*=0.93; 90-day mortality 1.00 (0.94–1.08), *P*=0.93. In 876 pairs of patients after PSM (ALBI grade 1 and grade 3), diabetes was also demonstrated to be uninfluential (in-hospital mortality, OR 0.85 (0.63, 1.12), *P*=0.25; 30-day mortality, HR 0.95 (0.75, 1.19), *P*=0.65; and 90-day mortality, 0.98 (0.79, 1.20), *P*=0.84. Similar trend was also observed in 2,232 pairs of patients (ALBI grade 1 and grade 2). Overall, the influence of diabetes on mortality of AKI is more likely to be minor. Although mortality of kidney failure due to diabetes mellitus was reported to be significantly higher than kidney failure attributed to AKI [25], we tend to consider diabetes as an uninfluential factor in our cohort.

CKD and AKI interact with each other [26]. Patients with CKD were reported to be more vulnerable to onset of AKI [27]. AKI also increased the possibility of progressing to be CKD or even end-stage renal disease (ESRD) [2, 28]. Intuitively, AKI patients with CKD will have poorer prognosis than those with normal renal function baseline. Actually, patients with CKD who develop AKI often recover incompletely and experience aggravated subsequent renal function decline [2, 29]. In our multivariate logistic and Cox regression, CKD seems not to be associated with mortality of AKI. However, in univariate regression, CKD is a strong risk factor for mortality (in-hospital mortality, OR 1.28, 95% CI (1.19–1.38), *P* < 0.001; 30-day mortality, HR 1.21 (1.12–1.32), *P* < 0.001; and 90-day mortality 1.28 (1.19–1.38), *P* < 0.001). After PSM, CKD remains a risk factor for 30-day mortality and 90-day mortality, but not in-hospital mortality (in-hospital mortality, OR 1.21, 95% CI (0.90–1.62), *P*=0.19; 30-day mortality, HR 1.28 (1.01–1.62), *P*=0.043; and 90-day mortality 1.29 (1.04–1.58), *P*=0.018); in 876 pairs of ALBI grade 1 and grade 3 patients with AKI. In 2,232 pairs of patients (ALBI grade 1 and grade 2), CKD is still a risk factor for 30-day mortality and 90-day mortality. The evidence in our study is inclined to support that the complication with CKD will worsen the clinical outcomes of AKI patients.

Propensity score matching was usually utilized to depress selection bias in clinical studies [30]. Previous study also utilized PSM in retrospective correlation study [31]. In this paper, we adopt PSM to match a few relevant covariates that may introduce bias and lead to false conclusion. Hepatic malignancy and cirrhosis which tend to influence the generation of albumin and bilirubin; sepsis, AMI, and other complications which may exert significant influence on mortality; SOFA, SAPSSII score, and a few blood biomarkers like lactate which will reflect the severity of illness. All above factors are included as adjusted covariates in PSM to reduce potential bias. The results in PSM are consistent with the conclusion drawn from K–M analysis and Cox regression.

ALBI grade could reflect systematic inflammation response. The elevated ALBI grade may hint overactive inflammation response in AKI patients [10], thus making aggressive usage of glucocorticoid a potential treatment option. However, such a proposal requires further validation in prospective trial. Glucocorticoids will also unavoidably cause harmful liquid and sodium reservation in body. Additionally, since elevated initial ALBI grade hints higher mortality, a few advanced therapies, like CRRT or other life support should be taken more aggressively. Furthermore, as an element of ALBI, albumin could modulate inflammation reaction and organic oxidation resistance [32]. Serum albumin will be consumed rapidly in severe inflammation due to oxidation, glycation, and relevant catabolism [32, 33]. Immediate albumin administration may be of help in improving prognosis [33]. However, we also have to note that albumin replacement in addition to crystalloids will not improve the rate of survival in patients with severe sepsis [34].

There are also some limitations in this retrospective study. There are repeated tests and records of blood albumin and bilirubin for a patient in MIMIC IV 2.0 database. However, we only utilize the initial value to calculate ALBI grade. Dynamic change during the whole pathology and illness course may be ignored. Furthermore, the data are all from one single center, which will unavoidably introduce bias.

## Figures and Tables

**Table 1:** Baseline of patients in cohorts.

Characteristics	ALBI grade 1	ALBI grade 2	ALBI grade 3	*P*
*n*	2,878	6,708	2,932	—
ALBI, mean (SD)	−2.94 (0.25)	−2.03 (0.34)	−0.90 (0.38)	<0.001
Age (years)	66.74 (15.87)	68.06 (16.22)	62.53 (15.68)	<0.001
Gender *n* (%)	1,704 (59.2)	3,745 (55.8)	1,734 (59.1)	0.001
Comorbidities *n* (%)				
Malignant neoplasm of liver	5 (0.2)	43 (0.6)	104 (3.5)	<0.001
Other malignancy	169 (5.9)	989 (14.7)	704 (24.0)	<0.001
Cirrhosis of liver	65 (2.3)	586 (8.7)	986 (33.6)	<0.001
Sepsis	216 (7.5)	1,715 (25.6)	1,429 (48.7)	<0.001
AMI	178 (6.2)	555 (8.3)	111 (3.8)	<0.001
HF	720 (25.0)	2,444 (36.4)	602 (20.5)	<0.001
Hypertension	808 (28.1)	1,643 (24.5)	620 (21.1)	<0.001
COPD	132 (4.6)	492 (7.3)	144 (4.9)	<0.001
CKD	566 (19.7)	1,784 (26.6)	533 (18.2)	<0.001
AF	982 (34.1)	2,405 (35.9)	761 (26.0)	<0.001
Cerebral infarction	255 (8.9)	437 (6.5)	89 (3.0)	<0.001
Cerebral hemorrhage	243 (8.4)	287 (4.3)	36 (1.2)	<0.001
Acute respiratory failure	609 (21.2)	2,350 (35.0)	1,251 (42.7)	<0.001
Rheumatic disease	82 (2.8)	262 (3.9)	103 (3.5)	0.038
Diabetes	883 (30.7)	2,201 (32.8)	775 (26.4)	<0.001
Laboratory parameters maximum, mean (SD)				
AST (U/L)	74.61 (355.23)	306.58 (1430.44)	646.61 (2133.29)	<0.001
ALP (U/L)	86.12 (35.46)	105.97 (90.09)	174.07 (202.37)	<0.001
ALT (U/L)	44.20 (223.21)	198.85 (1163.63)	323.20 (1048.90)	<0.001
Lactate (mmol/L)	2.66 (1.75)	2.85 (2.47)	4.03 (3.46)	<0.001
WBC (10^9^/L)	14.10 (8.37)	15.11 (11.45)	17.02 (13.97)	<0.001
Potassium (mEq/L)	4.66 (0.91)	4.72 (0.94)	4.72 (0.94)	0.017
Sodium (mEq/L)	140.11 (4.79)	140.04 (5.72)	139.09 (6.31)	<0.001
Glucose (mg/dL)	166.91 (108.43)	185.63 (120.75)	183.50 (105.96)	<0.001
Creatinine (mEq/L)	1.63 (2.21)	1.98 (2.14)	2.08 (1.79)	<0.001
BUN (mg/dL)	25.69 (22.46)	34.98 (26.74)	38.88 (28.61)	<0.001
INR	1.40 (0.77)	1.74 (1.48)	2.13 (1.46)	<0.001
Laboratory parameters minimum, mean (SD)				
Calcium (mg/dL)	8.36 (0.80)	7.98 (0.85)	7.47 (1.02)	<0.001
Platelet (10^9^/L)	181.57 (78.54)	185.55 (105.87)	139.76 (114.03)	<0.001
Hemoglobin (g/dL)	10.77 (2.25)	10.00 (2.24)	8.92 (2.06)	<0.001
AKI stage *n* (%)	—	—	—	<0.001
1	851 (29.6)	1,511 (22.5)	424 (14.5)	—
2	1,476 (51.3)	3,105 (46.3)	1,097 (37.4)	—
3	551 (19.1)	2,092 (31.2)	1,411 (48.1)	—
SAPSII score	34.60 (12.28)	40.83 (14.14)	47.69 (16.15)	<0.001
SOFA score	4.91 (3.01)	6.62 (3.81)	9.90 (4.45)	<0.001
Ventilation, *n* (%)	2,372 (82.4)	5,730 (85.4)	2,485 (84.8)	0.001
RRT, *n* (%)	134 (4.7)	431 (6.4)	323 (11.0)	<0.001
ALBI quartile	—	—	—	<0.001
0%–25%	2,878 (100.0)	235 (3.5)	0 (0.0)	—
25%–75%	0 (0.0)	6,273 (93.5)	0 (0.0)	—
75%–100%	0 (0.0)	200 (3.0)	2,932 (100.0)	—
In-hospital mortality, *n* (%)	244 (8.5)	1,194 (17.8)	949 (32.4)	<0.001
30-day mortality, *n* (%)	310 (10.8)	1,530 (22.8)	1,098 (37.4)	<0.001
90-day mortality, *n* (%)	404 (14.0)	1,984 (29.6)	1,332 (45.4)	<0.001

AMI, acute myocardial infarction; HF, heart failure; COPD, chronic obstructive pulmonary disease; CKD, chronic kidney disease; AF, atrial fibrillation; AST, aspartate transaminase; ASP, alkaline phosphatase; ALT, alanine transaminase; WBC, white blood cell count; BUN, blood urea nitrogen; INR, international normalized ratio; SAPSII, Simplified Acute Physiology Score II; SOFA, Sequential Organ Failure Assessment score; RRT, renal replacement therapy.

**Table 2:** Characteristics of patients in cohorts after PSM.

Characteristics	ALBI grade 1	ALBI grade 2	*P*	ALBI grade 1	ALBI grade 3	*P*
*n*	2,232	2,232	—	876	876	—
ALBI (mean (SD))	−2.93 (0.25)	−2.11 (0.34)	<0.001	−2.91 (0.25)	−1.03 (0.32)	<0.001
Age	67.48 (15.74)	67.29 (16.79)	0.701	66.45 (15.99)	65.18 (16.41)	0.1
Gender *n* (%)	1,290 (57.8)	1,311 (58.7)	0.544	501 (57.2)	497 (56.7)	0.885
Malignant neoplasm of liver, *n* (%)	5 (0.2)	5 (0.2)	1	5 (0.6)	8 (0.9)	0.578
Other malignancy, *n* (%)	160 (7.2)	147 (6.6)	0.478	122 (13.9)	130 (14.8)	0.634
Cirrhosis, *n* (%)	64 (2.9)	58 (2.6)	0.646	64 (7.3)	63 (7.2)	1
sepsis, *n* (%)	203 (9.1)	215 (9.6)	0.572	192 (21.9)	227 (25.9)	0.057
AMI, *n* (%)	151 (6.8)	136 (6.1)	0.393	69 (7.9)	43 (4.9)	0.015
COPD, *n* (%)	125 (5.6)	111 (5.0)	0.385	48 (5.5)	47 (5.4)	1
CKD, *n* (%)	488 (21.9)	466 (20.9)	0.443	232 (26.5)	184 (21.0)	0.008
Acute respiratory failure, *n* (%)	539 (24.1)	532 (23.8)	0.833	278 (31.7)	289 (33.0)	0.61
Lactate (mean (SD))	2.69 (1.79)	2.63 (1.89)	0.294	3.09 (2.45)	3.20 (2.35)	0.354
ALT (mean (SD))	48.91 (252.55)	61.76 (266.04)	0.098	81.80 (397.00)	87.84 (173.15)	0.68
ASTS (mean (SD))	81.30 (401.31)	88.76 (696.97)	0.107	130.94 (633.58)	147.16 (339.07)	0.504
ALP (mean (SD))	88.20 (37.83)	87.08 (50.42)	0.401	95.93 (52.45)	101.34 (73.50)	0.076
Creatinine (mean (SD))	1.68 (2.18)	1.63 (1.79)	0.409	2.30 (3.32)	1.76 (1.79)	<0.001
BUN (mean (SD))	27.13 (23.74)	26.78 (19.15)	0.593	33.80 (30.43)	30.88 (24.24)	0.027
SAPSII (mean (SD))	35.94 (12.36)	35.84 (12.11)	0.77	40.85 (13.78)	41.06 (13.54)	0.756
SOFA (mean (SD))	5.24 (3.05)	5.19 (3.23)	0.594	6.69 (3.61)	7.01 (3.71)	0.069
AKI stage, *n* (%)	—	—	0.939	—	—	0.542
1	620 (27.8)	627 (28.1)	—	217 (24.8)	199 (22.7)	—
2	1,135 (50.9)	1,137 (50.9)	—	407 (46.5)	410 (46.8)	—
3	477 (21.4)	468 (21.0)	—	252 (28.8)	267 (30.5)	—
Ventilation, *n* (%)	1,875 (84.0)	1,873 (83.9)	0.967	750 (85.6)	738 (84.2)	0.463
RRT, *n* (%)	104 (4.7)	112 (5.0)	0.625	79 (9.0)	57 (6.5)	0.061
In-hospital mortality, *n* (%)	213 (9.5)	270 (12.1)	0.007	122 (13.9)	158 (18.0)	0.026
30-day mortality, *n* (%)	270 (12.1)	368 (16.5)	<0.001	151 (17.2)	186 (21.2)	0.039
90-day mortality, *n* (%)	352 (15.8)	464 (20.8)	<0.001	197 (22.5)	245 (28.0)	0.01

**Table 3:** Aggregation of Cox regression models.

ALBI grade	Grade 1	Grade 2				Grade 3			
In-hospital death		OR	95% CI		*P*	OR	95% CI		*P*
Univariate	1	2.34	2.02	2.70	<0.001	5.17	4.44	6.02	<0.001
Model 1	1	2.29	1.99	2.66	<0.001	5.63	4.83	6.57	<0.001
Model 2	1	1.70	1.46	1.99	<0.001	3.18	2.67	3.79	<0.001
Model 3	1	1.31	1.11	1.55	0.001	1.52	1.24	1.85	<0.001
30-day death	—	HR	95% CI		*P*	HR	95% CI		*P*
Univariate	1	2.23	2.00	2.56	<0.001	4.14	3.65	4.70	<0.001
Model 1	1	2.22	1.96	2.51	<0.001	4.61	4.06	5.24	<0.001
Model 2	1	1.74	1.54	1.98	<0.001	2.81	2.49	3.29	<0.001
Model 3	1	1.43	1.26	1.63	<0.001	1.61	1.39	1.88	<0.001
90-day death	—	HR	95% CI		*P*	HR	95% CI		*P*
Univariate	1	2.31	2.07	2.57	<0.001	4.06	3.63	4.54	<0.001
Model 1	1	2.26	2.03	2.52	<0.001	4.59	4.11	5.14	<0.001
Model 2	1	1.76	1.58	1.97	<0.001	2.81	2.49	3.18	<0.001
Model 3	1	1.49	1.34	1.68	<0.001	1.71	1.49	1.95	<0.001

Model 1 included age and gender as covariates. Model 2 adjusted for covariates in Model 1 plus comorbidities including: malignant neoplasm of liver, other malignancy, cirrhosis of liver, sepsis, acute myocardial infarction (AMI), heart failure (HF), hypertension, chronic obstructive pulmonary disease (COPD), chronic kidney disease (CKD), atrial fibrillation (AF), cerebral infarction, cerebral hemorrhage, acute respiratory failure, rheumatic disease, and diabetes. Model 3 adjusted for covariates in Model 2 plus laboratory tests like maximum of aspartate transaminase (AST), alkaline phosphatase (ASP), alanine transaminase (ALT), lactate, white blood cell (WBC) count, blood potassium, sodium, glucose, serum creatinine, blood urea nitrogen (BUN), international normalized ratio (INR); minimum of blood calcium, platelet count, and hemoglobin; SOFA and SPASII score, use of ventilation and RRT.

**Table 4:** Univariate Cox regression model 3 for patients after PSM.

Group	HR	95% CI lower limit	Upper limit	*P*
Grade 2				
In-hospital mortality	1.30	1.08	1.58	0.006
30-day mortality	1.39	1.19	1.63	<0.001
90-day mortality	1.36	1.18	1.56	<0.001
Grade 3				
In-hospital mortality	1.17	1.06	1.28	0.026
30-day mortality	1.22	1.11	1.35	0.039
90-day mortality	1.23	1.13	1.34	0.01

## Data Availability

All data can be acquired from public database: https://physionet.org/content/mimiciv/2.0/.

## Supplementary Materials

Supplementary 1 Table 1: results of all variables in multivariate Cox regression based on Model 3.

Supplementary 2 Table 2: aggregation of Cox regression models by quartiles of ALBI grade.
