# Supplementary material for: ALBI Grade Is Associated with Clinical Outcomes of Critically Ill Patients with AKI: A Cohort Study with Cox Regression and Propensity Score Matching
Source: Mediators Inflamm. 2024 Jul 18;2024:1412709. doi: 10.1155/2024/1412709 (PMC11272401; doi:10.1155/2024/1412709)
Supplement: Supplementary 1 — Table 1: results of all variables in multivariate Cox regression based on Model 3. [file 1412709.f1.pdf]

Multivariate Cox regression Model 3

**in_hospital_death**	Odds ratio	95% conf.	interval	P>z
ALBI grade				
2.00	1.30	1.10	1.54	<0.001
3.00	1.52	1.24	1.85	<0.001
Age	1.01	1.01	1.02	<0.001
gender	0.98	0.88	1.09	0.71
Comorbidities				
Sepsis	1.50	1.33	1.68	<0.001
AMI	1.39	1.15	1.70	<0.001
HF	0.83	0.73	0.94	<0.001
Cirrhosis	1.27	1.08	1.49	<0.001
Hypertension	0.98	0.86	1.12	0.80
COPD	1.03	0.84	1.27	0.77
CKD	0.94	0.81	1.09	0.39
AF	1.00	0.89	1.13	1.00
Cerebral infarction	1.35	1.09	1.67	0.01
Cerebral hemorrhage	3.73	2.98	4.68	<0.001
Acute respiratory failure	2.11	1.88	2.36	<0.001
Rheumatic_disease	1.14	0.87	1.49	0.35
Malignant neoplasm of liver	0.52	0.32	0.83	0.01
Other maligancy	1.45	1.25	1.67	<0.001
Diabetes	0.77	0.68	0.87	<0.001
Hemoglobin	0.98	0.85	1.14	0.82
WBC				
2.00	1.71	1.28	2.30	<0.001
3.00	1.11	0.98	1.25	0.10
Platelet	0.89	0.79	1.01	0.06
Potassium				
2.00	1.32	1.15	1.50	<0.001
3.00	1.34	1.02	1.76	0.04
Sodium				
2.00	1.16	1.02	1.32	0.02
3.00	1.21	0.92	1.59	0.16
Lactate	1.26	1.11	1.43	<0.001
Calcium	0.78	0.69	0.89	<0.001
INR	1.45	1.28	1.63	<0.001
ALT	1.09	0.96	1.25	0.19
ALP	1.34	1.17	1.53	<0.001
AST	1.03	0.90	1.18	0.64
Glucose	1.24	1.10	1.39	<0.001
Scr	0.93	0.80	1.07	0.32
BUN	1.22	1.05	1.41	0.01
AKI stage				
2.00	1.19	1.01	1.40	0.04
3.00	2.24	1.90	2.65	0.00
SOFA score	1.05	1.02	1.07	<0.001
SAPSII score	1.03	1.03	1.04	<0.001
Ventilation	1.17	0.99	1.39	0.07
RRT	0.76	0.63	0.92	0.01

**30-day death**	Hazards ratio	95% conf	interval	P>z
ALBI grade				
2.00	1.43	1.26	1.63	<0.001
3.00	1.61	1.39	1.88	<0.001
Age	1.02	1.01	1.02	<0.001
gender	0.99	0.92	1.07	0.858
Comorbidities				
Sepsis	1.17	1.08	1.27	<0.001
AMI	1.22	1.06	1.39	0.005
HF	0.87	0.80	0.95	0.002
Cirrhosis	1.28	1.15	1.44	<0.001
Hypertension	0.95	0.87	1.05	0.348
COPD	1.04	0.91	1.21	0.547
CKD	0.94	0.85	1.04	0.208
AF	0.88	0.81	0.96	0.003
Cerebral infarction	1.27	1.10	1.47	0.001
Cerebral hemorrhage	2.38	2.02	2.80	<0.001
Acute respiratory failure	1.41	1.30	1.54	<0.001
Rheumatic_disease	0.98	0.80	1.19	0.816
Malignant neoplasm of liver	0.79	0.59	1.07	0.124
Other maligancy	1.38	1.26	1.52	<0.001
Diabetes	0.85	0.78	0.93	<0.001
Hemoglobin	0.94	0.84	1.04	0.229
WBC				
2.00	1.37	1.12	1.67	0.002
3.00	1.12	1.03	1.22	0.011
Platelet	0.84	0.77	0.92	<0.001
Potassium				
2.00	1.19	1.08	1.31	<0.001
3.00	1.39	1.17	1.66	<0.001
Sodium				
2.00	1.18	1.08	1.29	<0.001
3.00	1.34	1.13	1.60	0.001
Lactate	1.21	1.10	1.32	<0.001
Calcium	0.85	0.77	0.93	<0.001
INR	1.43	1.31	1.55	<0.001
ALT	1.04	0.94	1.14	0.475
ALP	1.38	1.26	1.51	<0.001
AST	0.95	0.86	1.04	0.251
Glucose	1.10	1.01	1.19	0.023
Scr	0.98	0.88	1.09	0.736
BUN	1.26	1.12	1.41	<0.001
AKI stage				
2.00	1.25	1.10	1.42	<0.001
3.00	1.87	1.65	2.13	<0.001
SOFA score	1.02	1.01	1.04	0.001
SAPSII score	1.02	1.02	1.03	<0.001
Ventilation	1.12	0.99	1.27	0.063
RRT	0.81	0.71	0.92	0.001

**90-day death**	Hazards ratio	95% conf	interval	P>z
ALBI grade				
2.00	1.49	1.33	1.67	<0.001
3.00	1.71	1.49	1.95	<0.001
Age	1.02	1.02	1.02	<0.001
gender	1.00	0.94	1.07	0.97
Comorbidities				
Sepsis	1.24	1.15	1.34	<0.001
AMI	1.17	1.03	1.32	0.02
HF	0.95	0.88	1.03	0.23
Cirrhosis	1.39	1.25	1.53	<0.001
Hypertension	0.95	0.87	1.03	0.22
COPD	1.02	0.90	1.16	0.75
CKD	0.95	0.87	1.03	0.22
AF	0.89	0.83	0.96	<0.001
Cerebral infarction	1.33	1.18	1.51	<0.001
Cerebral hemorrhage	2.29	1.98	2.65	<0.001
Acute respiratory failure	1.39	1.29	1.50	<0.001
Rheumatic_disease	1.04	0.88	1.23	0.66
Malignant neoplasm of liver	0.79	0.61	1.03	0.09
Other maligancy	1.51	1.39	1.64	<0.001
Diabetes	0.88	0.82	0.95	<0.001
Hemoglobin	0.99	0.90	1.09	0.90
WBC				
2.00	1.28	1.07	1.53	0.01
3.00	1.07	0.99	1.15	0.10
Platelet	0.84	0.78	0.91	<0.001
Potassium				
2.00	1.19	1.10	1.30	<0.001
3.00	1.33	1.14	1.57	<0.001
Sodium				
2.00	1.21	1.12	1.31	<0.001
3.00	1.38	1.18	1.61	<0.001
Lactate	1.18	1.09	1.28	<0.001
Calcium	0.82	0.76	0.89	<0.001
INR	1.36	1.26	1.46	<0.001
ALT	0.98	0.90	1.07	0.72
ALP	1.47	1.36	1.60	<0.001
AST	0.93	0.85	1.01	0.09
Glucose	1.05	0.98	1.13	0.16
Scr	0.97	0.89	1.07	0.54
BUN	1.26	1.14	1.39	<0.001
AKI stage				
2.00	1.16	1.05	1.29	0.01
3.00	1.67	1.50	1.86	<0.001
SOFA score	1.02	1.01	1.03	<0.001
SAPSII score	1.02	1.02	1.03	<0.001
Ventilation	1.04	0.94	1.16	0.42
RRT	0.84	0.74	0.94	<0.001
