# Supplementary material for: ALBI Grade Is Associated with Clinical Outcomes of Critically Ill Patients with AKI: A Cohort Study with Cox Regression and Propensity Score Matching
Source: Mediators Inflamm. 2024 Jul 18;2024:1412709. doi: 10.1155/2024/1412709 (PMC11272401; doi:10.1155/2024/1412709)
Supplement: Supplementary 2 — Table 2: aggregation of Cox regression models by quartiles of ALBI grade. [file 1412709.f2.pdf]

ALBI quartile	Quartile 1		Quatile 2					Quartile 3
In-hospital death		OR	95%CI		P	OR	95%CI	
Univariate	1	2.24	1.94	2.57	<0.001	4.91	4.25	5.67
Model 1	1	2.19	1.91	2.53	<0.001	5.32	4.59	6.16
Model 2	1	1.63	1.41	1.89	<0.001	3.03	2.56	3.58
Model 3	1	1.23	1.05	1.45	0.011	1.44	1.19	1.75
30-day death		HR	95%CI		P	HR	95%CI	
Univarite	1	2.12	1.88	2.38	<0.001	3.87	3.43	4.36
Model 1	1	2.07	1.85	2.33	<0.001	4.27	3.79	4.81
Model 2	1	1.63	1.44	1.84	<0.001	2.66	2.33	3.04
Model 3	1	1.33	1.18	1.51	<0.001	1.51	1.31	1.74
90-day death								
Univarite	1	2.16	1.95	2.40	<0.001	3.79	3.41	4.24
Model 1	1	2.12	1.92	2.35	<0.001	4.24	3.81	4.72
Model 2	1	1.65	1.49	1.84	<0.001	2.61	2.32	2.93
Model 3	1	1.39	1.25	1.55	<0.001	1.58	1.39	1.79

P
<0.001
<0.001
<0.001
<0.001
P
<0.001
<0.001
<0.001
<0.001
<0.001
<0.001
<0.001
<0.001
